# Genome-wide identification, classification and expression profiling of *nicotianamine synthase* (*NAS*) gene family in maize

**DOI:** 10.1186/1471-2164-14-238

**Published:** 2013-04-10

**Authors:** Xiaojin Zhou, Suzhen Li, Qianqian Zhao, Xiaoqing Liu, Shaojun Zhang, Cheng Sun, Yunliu Fan, Chunyi Zhang, Rumei Chen

**Affiliations:** 1 Department of Crop Genomics & Genetic Improvement, Biotechnology Research Institute, Chinese Academy of Agricultural Sciences, Beijing 100081, China; 2 National Key Facility for Crop Gene Resources and Genetic Improvement (NFCRI), Beijing 100081, China; 3 Department of Agronomy, Agricultural University of Hebei/Hebei Sub-center of Chinese National Maize Improvement Center, Baoding 071001, China

**Keywords:** Maize, Nicotianamine synthase, Gene family, Iron uptake and homeostasis, Subcellular localization, Expression profiling, *In situ* hybridization

## Abstract

**Background:**

Nicotianamine (NA), a ubiquitous molecule in plants, is an important metal ion chelator and the main precursor for phytosiderophores biosynthesis. Considerable progress has been achieved in cloning and characterizing the functions of nicotianamine synthase (NAS) in plants including barley, *Arabidopsis* and rice. Maize is not only an important cereal crop, but also a model plant for genetics and evolutionary study. The genome sequencing of maize was completed, and many gene families were identified. Although three *NAS* genes have been characterized in maize, there is still no systematic identification of maize *NAS* family by genomic mining.

**Results:**

In this study, nine *NAS* genes in maize were identified and their expression patterns in different organs including developing seeds were determined. According to the evolutionary relationship and tissue specific expression profiles of *ZmNAS* genes, they can be subgrouped into two classes. Moreover, the expression patterns of *ZmNAS* genes in response to fluctuating metal status were analysed. The class I *ZmNAS* genes were induced under Fe deficiency and were suppressed under Fe excessive conditions, while the expression pattern of class II genes were opposite to class I. The complementary expression patterns of class I and class II *ZmNAS* genes confirmed the classification of this family. Furthermore, the histochemical localization of *ZmNAS1;1/1;2* and *ZmNAS3* were determined using *in situ* hybridization. It was revealed that *ZmNAS1;1/1;2,* representing the class I genes, mainly expressed in cortex and stele of roots with sufficient Fe, and its expression can expanded in epidermis, as well as shoot apices under Fe deficient conditions. On the contrary, *ZmNAS3*, one of the class II genes, was accumulated in axillary meristems, leaf primordia and mesophyll cells. These results suggest that the two classes of *ZmNAS* genes may be regulated on transcriptional level when responds to various demands for iron uptake, translocation and homeostasis.

**Conclusion:**

These results provide significant insights into the molecular bases of *ZmNAS* in balancing iron uptake, translocation and homeostasis in response to fluctuating environmental Fe status.

## Background

Iron is an essential micronutrient with numerous cellular functions in animals and plants. The anemia caused by iron-deficiency is still a prevalent nutrient problem affecting more than half of the world’s population, especially in developing countries [1]. Besides, iron is also an essential metal nutrient factor for plants, as it plays critical roles during many development processes, including photosynthesis, respiration, and other biochemical reactions that need Fe as a co-factor. Iron deficiency in plants may lead to leaf senescence, and in turn severely reduced the yield and quality. The total amount of Fe in soil is not limited; however, it can be merely soluble under aerobic conditions, especially in alkaline and calcareous soil [2]. In order to acquire enough Fe without toxicity, plants have developmented iron uptake, utilization and storage system regulated by environmental Fe availability. The mechanism of Fe acquisition in plants can be divided into two categories: strategy I and strategy II [3]. The strategy I was applied by nongraminaceous plants, which includes the reduction of ferric to ferrous on the root surface, and absorption of ferrous across the root plasma membrane by Fe^2+^ transporters. The FRO2 [4] and IRT1 [5] were firstly cloned from *Arabidopsis* and responsible for these processes. The graminaceous plants, such as rice, corn and barley, applied strategy II, which includes the synthesis and secretion of mugineic acid (MAs) family phytosiderophores (PS) from roots and the uptake of Fe^3+^-PS complexes by specific plasma membrane transporters. MAs can be synthesized by a conserved pathway begin with trimerization of three molecular of S-adenosyl-L-methionine into nicotianamine (NA) by nicotianamine synthase (NAS) [6], and then NA is converted into 2^′^-deoxymugineic acid (DMA), the precursor of MAs, by nicotianamine aminotransferase (NAAT) [7] and deoxymugineic acid synthase (DMAS) [8]. In some graminaceous plants MAs can be obtained by hydroxylation of DMA [9,10]. NA is known as a metal chelator, which can bind a range of metals, including Fe, Zn, Mn and Cu [11-15]. When iron was absorbed in plants, its translocation is thought to be associated with appropriate chelators, such as citrate [16,17], NA [1,14], and MAs [18,19]. Citrate is essential in Fe transportation in xylem sap [16], while NA play a dominant role in the chelating and trafficking of Fe in phloem [20]. In graminaceous plants, yellow strip like (YSL) family transporter, YS1, was reported facilitating the Fe^3+^-DMA uptake from rhizosphere [21], while AtYSL1 and AtYSL3 involved in long-distance translocation of Fe^2+^-NA in nongraminaceous plants [20,22-24]. A tomato NA synthesis mutant, *chloronerva* (*chln*), show phenotype defects in Fe utilization and homeostasis [25,26]. In addition, transgenic tobacco plants that continuously expressed barley NAAT exhibited disorders in internal metal transport, such as interveinal chlorosis in young leaves and abnormally shaped and sterilized flowers [14]. In the NAAT tobacco, the endogenous NA was consumed as a result of excessive produced NAAT, suggesting that NA play critical roles in the regulation of metal transfer in plants, and maintaining sufficient amount of NA is required for inner metal homeostasis. A recent study reported that activation of *OsNAS3* resulted in elevated Fe and Zn content in both vegetative tissue and seeds. Anemic mice fed with the *OsNAS3* activated rice recovered more rapidly than those with wild type rice. Moreover, activated *OsNAS3* expression also leads to increased tolerance to both Fe/Zn deficiencies and heavy-metal toxicity [27]. This report suggested that NA is critical for Fe acquisition and storage, as well as detoxification of excessive intracellular Fe in plants.

Maize (*Zea mays*) is a major crop plant for feed industry and food, as well as a research model for monocotyledon plant. Although the iron content in corn is relatively higher than that in brown rice [28], it can barely meet the increasing demand for feed production. Therefore, investigating the mechanisms of iron acquisition, translocation and homeostasis in maize may support a model for understanding that in other crop plants, and provide gene resources for further breeding maize varieties with enhanced iron content. Since NA is the key for regulating Fe homeostasis in plants, considerable progress has been achieved in cloning and characterizing the functions of NAS in plants, including barley [29,30], *Arabidopsis*[31], rice [32], tomato [25] and maize [33]. Although it has been demonstrated that NA facilitate iron acquisition and translocation by forming Fe^2+^–NA complexes and serving as the precursor of MAs, the mechanism regulating these two pathways under fluctuating environmental iron status is still unclear. Systematic analyses in *NAS* gene families revealed that there are three *NAS* genes in rice and four in *Arabidopsis*, which suggested that NAS are encoded by a few genes instead of a gene family [31,34]. However, nine *NAS* members were mapped in barley by combined approaches [30]; and it was also suggested that there are five genes encode NAS in maize, though only three of them were cloned due to the lack of genome information [33]. The relatively larger numbers of *NAS* genes in barley and maize indicates that *NAS* may duplicate and evolve during the emergence of new species and breeding process.

The maize genome had been thoroughly sequenced and assembled recently, whereas there is still no systematic identification and characterization of *NAS* family. To better understand the roles of *ZmNAS* genes in iron uptake, translocation and homeostasis, the sequences encoding NAS were analyzed by searching the maize inbred line B73 genome database. Nine similar sequences encoding putative NAS family members were explored. In this study, we provided detailed information on the phylogeny, subcellular localization, expression patterns and histochemical localization of the family. In particular, the ZmNAS family was subgrouped into class I and II depending on the phylogenetic relationship between graminaceous and nongraminaceous plants. Moreover, a comparison of the expression in different tissues and under various metal status provides further evidence for the specialization of *ZmNAS* in iron acquisition and homeostasis.

## Results

### Identification and cloning of *ZmNAS* genes

To detect all members of the *ZmNAS* family in the maize genome, a systematic TBLASTN search against the maize (B73) genome database was performed using protein sequence of ZmNAS1 as a query. Based on an e-value threshold of 10^-80^ and the present of the intact NAS domain, nine genes encoding putative ZmNAS were identified (Table 1), including three published genes, *ZmNAS1* (*ZmNAS1;1*), *ZmNAS2* (*ZmNAS2;1*) and *ZmNAS3*. The additional genes were named based on the similarity between previously identified ZmNASs (Additional file 1) and their positions in 10 chromosomes. Among the predicted *ZmNASs*, three of them (*ZmNAS4*, *ZmNAS5* and *ZmNAS6;1*) were confirmed by RT-PCR cloning and sequencing. Since the *ZmNAS1;1*/*ZmNAS1;2, ZmNAS2;1*/*ZmNAS2;2* and *ZmNAS6;1*/*ZmNAS6;2* share high similarity even in the 3^′^-untranslated region (Additional file 2) and the *ZmNASs* are intron-less, *ZmNAS1;2*, *ZmNAS2;2* and *ZmNAS6;2* were cloned from maize genomic DNA. Motif scan in Pfam database (http://pfam.sanger.ac.uk/) confirmed that all ZmNAS proteins contain an intact NAS domain, except ZmNAS2;1 and ZmNAS2;2 contain two full length NAS domains in tandem position.

**Table 1:** **BLAST analysis for the maize *Nicotianamine Synthase *genes ( *ZmNAS *) based on the genome database**

**Designated**			**Maize genome**		
**Given name (previous name)**	**cDNA**	**Chromosome no.**	**Genomic locus (bp)**	**cDNA length (bp)**	**Amino acids**
*ZmNAS1;1* (*ZmNAS1*)	GRMZM2G385200	9	135,550,861-135,552,092	1232	327
	GRMZM2G034956	9	135,796,454-135,797,695	1242	327
*ZmNAS1;2*	GRMZM2G312481	9	135,720,514-135,721,745	1232	327
*ZmNAS2;1* (*ZmNAS2*)	GRMZM2G030036	1	49,287,309-49,289,760	2452	601
*ZmNAS2;2*	GRMZM2G124785	1	49,320,819-49,323,269	2451	601
*ZmNAS3*	GRMZM2G478568	1	259,776,858-259,778,542	1685	359
*ZmNAS4*	GRMZM2G439195	5	15,799,611-15,801,603	1993	356
*ZmNAS5*	GRMZM2G050108	7	174,402,882-174,404,870	1989	422
*ZmNAS6;1*	GRMZM2G704488	9	135,299,182-135,300,357	1176	327
*ZmNAS6;2*	AC233955.1_FGT003	9	135,306,483-135,307,466	984	327

**Figure 1:**
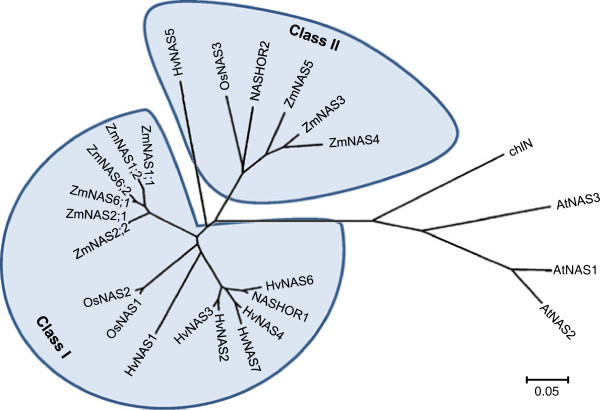
**Neighbor-joining phylogenetic tree of the NAS members.** The tree was constructed with the amino acid sequences of NAS proteins from Maize (Zm), Barley (Hv), Rice (Os), *Arabidopsis thaliana* (At) and *Solanum lycopersicum* (chlN) using the neighbor-joining method in MEGA 4.0 software. For proteins and accession numbers used in phylogenetic analysis, refer to “Methods”. The scale bar corresponds to a distance of 5 changes per 100 amino acid positions.

**Figure 2:**
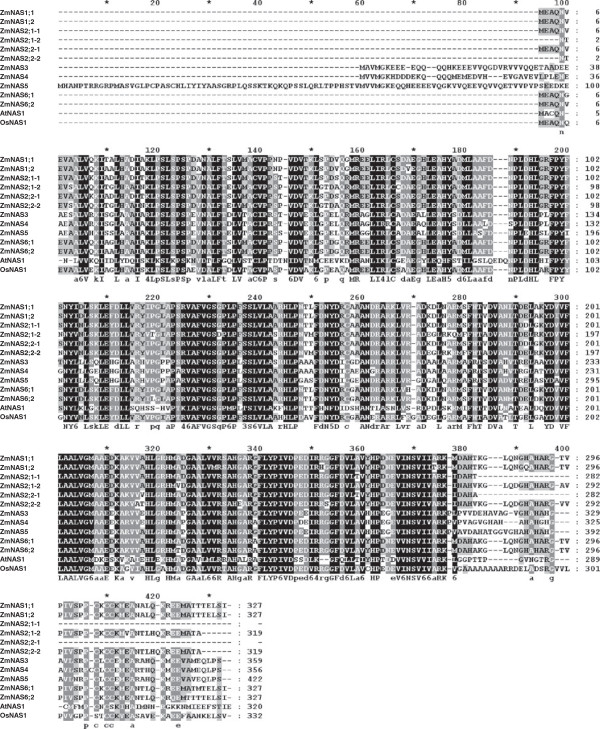
**The multiple sequence alignment of maize NAS members with AtNAS1 from *Arabidopsis thaliana *and OsNAS1 from rice.** The first and second NAS region in ZmNAS2;1 (ZmNAS2;2) were designed as ZmNAS2;1–1 (ZmNAS2;2–1) and ZmNAS2;1–2 (ZmNAS2;2–2), respectively. The light or dark shaded backgrounds indicate partial or entire conservative residues.

To understand the link between the function and evolutionary relationship of ZmNASs, the phylogenetic tree between all NASs from maize, barley, rice, *Arabidopsis* and tomato was established by the neighbor-joining method (Figure 1). The result shows that there exists a divergence between graminaceous and nongraminaceous plants, and the NASs from graminaceous plants were distinctly divided into two groups, class I and class II. In addition, it can be found that there are relatively more class I *NAS* genes existed in maize and barley than in rice. It was also shown that the class I *ZmNAS* genes fall into sister pairs, *ZmNAS1;1*/*ZmNAS1;2*, *ZmNAS2;1*/*ZmNAS2;2* and *ZmNAS6;1*/*ZmNAS6;2*, and the duplication of *ZmNAS* was associated with the chromosomal block duplications [35]. Moreover, The ZmNAS paralogs were closer to each other than their orthologs in barley and rice, indicating that these gene pairs arose during the whole genome duplication after the divergence from the common ancestor of maize and barley. Since NA is an important metal-chelator in plants, it can be assume that NAS family enlarged to meet the increasing demand for iron in maize and barley during environmental pressures and artificial breeding. The protein sequences of ZmNASs were aligned with AtNAS1 and OsNAS1, and the tandem domains of ZmNAS2;1 and ZmNAS2;2 were separated and aligned as part1 and part2 (Figure 2). This result revealed that all ZmNASs contain a highly conserved NAS domain of about 280 amino acid residues, including the two parts of ZmNAS2;1 and ZmNAS2;2. In addition, it is worthy to note that the class II ZmNASs contain a variable N-terminal domain, which may associated with their physiological function or subcellular localization.

### Subcellular localization of ZmNASs

**Figure 3:**
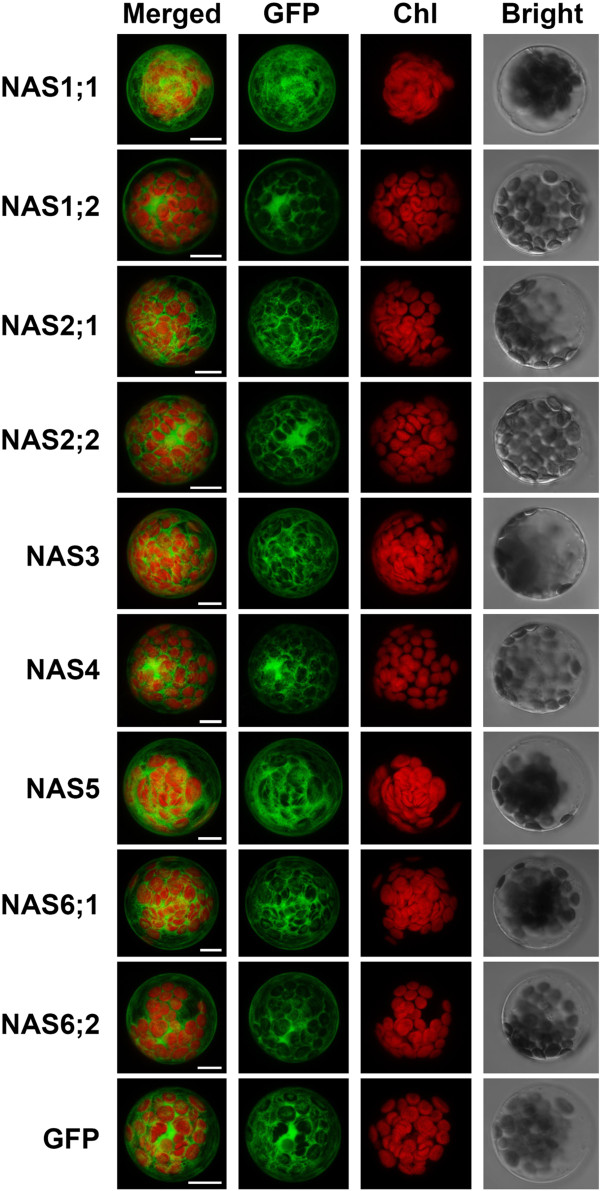
**Subcellular localization of ZmNAS-GFP fusion proteins in *Arabidopsis *mesophyll protoplasts*.*** The coding regions of *ZmNAS* genes were C-terminal fused with GFP and were transiently expressed in *Arabidopsis* mesophyll protoplasts. The GFP signal is shown in green and chlorophyll autofluorescence (Chl) is indicated in red. The images were obtained by a confocal microscope, and the cytoplasm localization of GFP is used as a control. The scale bar represents 10 μm.

To verify whether the N-terminal variable domain of class II ZmNASs may determine their specific subcellular localization, the coding regions of *ZmNASs* were C-terminal fused with green fluorescent protein (GFP) and the fusion protein was expressed under cauliflower mosaic virus 35S promoter. Then the resulting plasmids were transformed into *Arabidopsis* mesophyll protoplasts, respectively. The fluorescence of all ZmNAS-GFP was detected in the cytoplasm, which is similar to that of GFP-transformed cells (Figure 3). This result revealed that ZmNAS localized in cytoplasm, indicating that the specific N-terminal domain of class II ZmNASs and the phylogenetic difference between two classes of ZmNASs do not interfere with their subcellular localization.

### Complementary expression patterns of class I and class II *ZmNAS* genes

**Figure 4:**
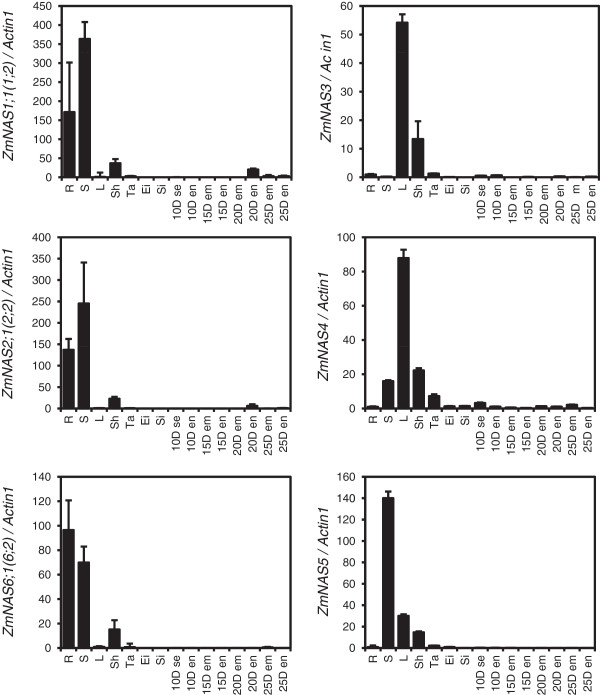
**Relative expression of *ZmNAS *genes in different mazie organs.** Total RNA was extracted from root (R), stem (S), leaf (L), leaf sheath (Sh), tassel (Ti), immature ear (Ei), silk (Si), as well as seed (se), endosperm (en) and embryo (em) at indicated days after pollination (10D-25D). For each gene, the relatively expression levels were obtained by normalization with maize *Actin1*. The error bars indicate standard deviations.

Although the two classes of ZmNASs shared identical subcellular localization, we hypothesized they may be differentially regulated in expression. Therefore, to analyse the physiological functions of ZmNAS in iron uptake, translocation and storage, their mRNA accumulation patterns were examined by quantitative reverse transcription PCR in various organs and developing seeds, with maize *Actin1* as an internal control (Figure 4). Since, the class I *ZmNAS* genes share high sequence similarity even in the 3’-untranslated region (Additional file 2), they were detected as sister pairs: *ZmNAS1;1*/*ZmNAS1;2*, *ZmNAS2;1*/*ZmNAS2;2* and *ZmNAS6;1*/*ZmNAS6;2*. The results showed that *ZmNAS* genes were merely accumulated in reproductive organs and exhibited complementary expression patterns in vegetative organs. The class I *ZmNAS* genes predominantly expressed in roots and stems; whereas class II were mainly accumulated in leaves and sheaths, with the exception of *ZmNAS5,* which was abundantly expressed in stems. This result suggested that class I *ZmNAS* genes may be involved in the Fe uptake in roots and long distance translocation in stems, while class II may contribute to the local transportation of Fe.

**Figure 5:**
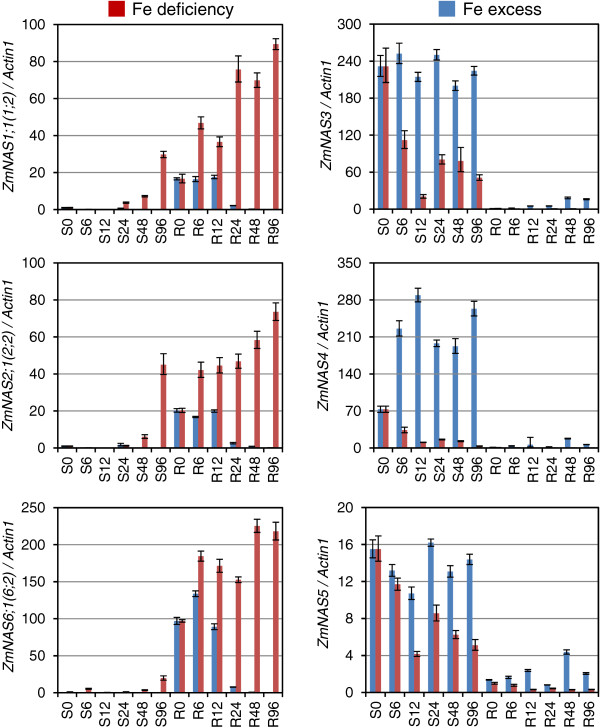
**Expression profiles of *ZmNAS *genes in response to Fe deficiency and Fe excess.** The maize seedlings were hydroponically cultured to three-leaf stage, and then they were transferred to Hoagland solution in the absence of Fe (Fe deficiency) or with 500 μM Fe^3+^-EDTA (Fe excess). The shoots (S) and roots (R) were harvested after 0, 6, 12, 24, 48 and 96 hours of treatment. Relative gene expressions were normalized using maize *Actin1*. The error bars indicate standard deviations.

**Figure 6:**
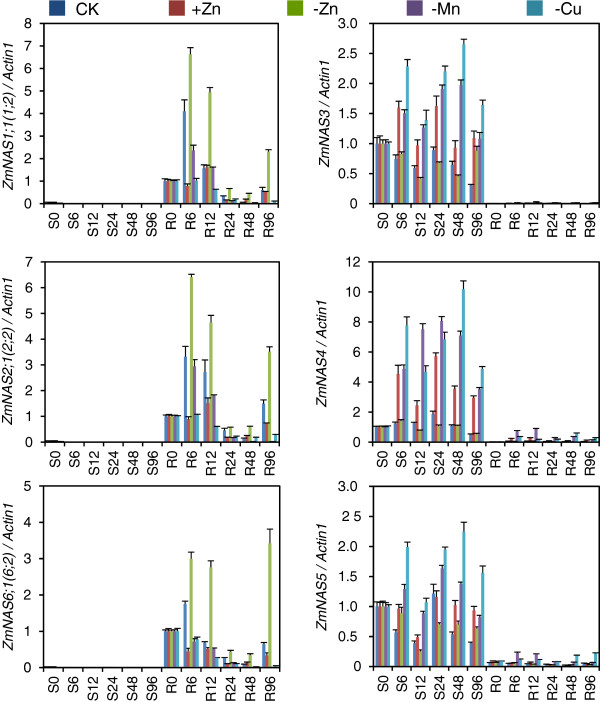
**Expression patterns of *ZmNAS *genes under various environmental Zn, Cu and Mn status.** The maize seedlings were hydroponically cultured to three-leaf stage, and then they were transferred to Hoagland solution with 200 μM ZnSO_4_ or without indicated metals. The shoots (S) and roots (R) were harvested after 0, 6, 12, 24, 48 and 96 hours of treatment. Relative genes expressions were normalized using maize *Actin1*. The error bars indicate standard deviations.

The expression of *ZmNAS* genes in seedlings subjected to Fe deficient and excessive conditions were investigated (Figure 5). The transcripts of class I *ZmNAS* genes were dramatically induced by Fe deficiency and were suppressed by Fe excess in both shoots and roots. On the contrary, the accumulation of class II *ZmNAS* genes were down-regulated by Fe deficiency in both shoots and roots, while they were up-regulated in roots in response to Fe excess. In addition, the expression level of *ZmNAS3* and *ZmNAS5* remained at a high level in shoots under excessive Fe status, though that of *ZmNAS4* was induced. Since NA can chelate various metals [20,36,37], the expression profiles of *ZmNAS* genes in response to other metal conditions were investigated (Figure 6). The result revealed that class I *ZmNAS* genes were stimulated under Zn deficiency, while they were suppressed in response to Zn excess and Cu/Mn deficiency. In contrast, the class II genes were induced under excessive Zn and deficient Cu/Mn conditions. These results showed that class I and class II *ZmNAS* genes were independently regulated in transcription and have complementary expression patterns under the same metal nutrient condition, which suggested that they may have different physiological functions associated with the uptake, translocation and storage of metal ions.

### Histochemical localization of *ZmNAS* genes

**Figure 7:**
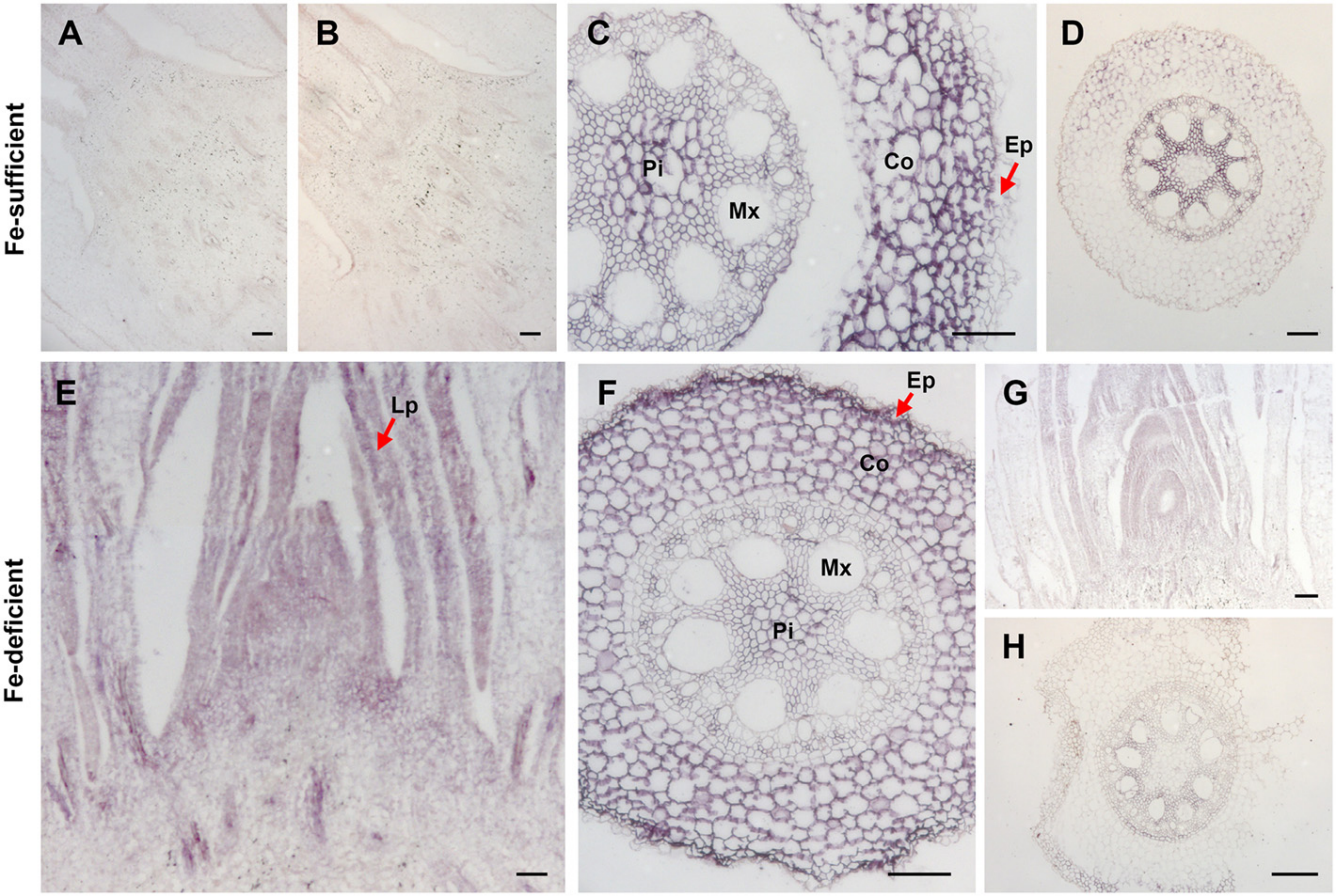
**Histochemical localization of *ZmNAS1;1/1;2 *in maize seedlings under Fe sufficient and deficient status.***In situ* hybridization was performed on shoot and root sections of maize seedlings under Fe sufficient (upper panel) and deficient (lower panel) conditions with digoxigenin-labeled antisense or sense probes. Longitudinal sections of shoot apex (**A, B, E** and **G**), and transverse sections of root (**C, D, F** and **H**) were hybridized. The expression of *ZmNAS1;1/1;2* was observed as purple staining in cortex and stele of Fe sufficient roots (**C**), epidermis, cortex and stele of Fe deficient roots (**F**), and leaf primordia of shoot apices (**E**) detected with antisense probes. No signal was observed in either control sections with sense probes (**B**, **D**, **G** and **H**) or shoot apices of Fe sufficient seedlings detected with antisense probes (**A**). Arrow indicates leaf primordia (**E**) and epidermis of roots (**C** and **F**). Pi, pith; Mx, Metaxylem; Co, cortex; Ep, epidermis; Lp, leaf primordia. The length of bars corresponds to 100 μm.

**Figure 8:**
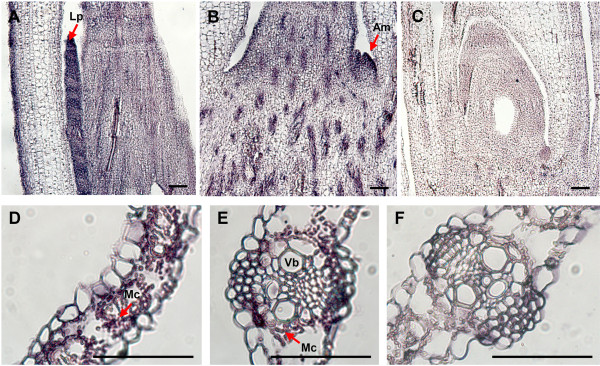
**Histochemical localization of *ZmNAS3 *in maize shoots.***In situ* hybridization was performed on longitudinal sections of shoot (**A, B** and **C**) and transverse sections of leaf (**E, D** and **F**) using digoxigenin-labeled antisense or sense probes. The hybridization signal was localized in the leaf primordia (**A**), axillary meristem (**B**) and mesophyll cells (**D** and **E**), while no signal was detectable in control sections hybridized with sense probes (**C** and **F**). Arrow indicates the hybridization signals represented by purple staining. Lp, leaf primordia; Am, axillary meristem; Mc, mesophyll cells; Vb, vascular bundles. The length of bars corresponds to 100 μm.

Histochemical localization of *ZmNAS* genes may help explain their expression patterns and putative roles in regulating NA biosynthesis in maize plants. In order to distinguish the tissue specific localization of the two classes of *ZmNAS* genes, the probes specifically recognize *ZmNAS1;1/1;2* or *ZmNAS3* were designed and synthesized. *In situ* hybridization showed that the signals of *ZmNAS1;1/1;2* were specifically detected in the cortex and stele of roots under Fe sufficient conditions (Figure 7C), whereas no signals can be observed in shoots (Figure 7A). In response to Fe deficiency, strong signals of *ZmNAS1;1/1;2* were also observed in epidermis of roots, and relatively weak signals were detected in leaf primordia (Figure 7F and 7E). These results suggested that maize roots respond to iron deficiency by inducing the spatially restricted expression of class I *ZmNAS*, which may lead to elevated NA biosyntheses and MAs secretion. Since, the expression analysis demonstrated that *ZmNAS3* was predominantly accumulated in leaves, *in situ* hybridization was performed using shoot apices and young leaves. The histochemical distribution of *ZmNAS3* was detected mainly in the leaf primordia and axillary meristems in shoot apices (Figure 8A and 8B) and mesophyll cells in young leaves (Figure 8D and 8E), suggesting that *ZmNAS3*, a member of class II *ZmNAS* genes, may participate in the local transportation and homeostasis of Fe in developing tissues.

## Discussion

### Identification of *ZmNAS* family

NAS was firstly identified in barley for catalyzing the trimerization of SAM into one molecule of NA [6], which is a key molecular chelating divalent metal ion and facilitating metal translocation in plants. In addition, NA is also the precursor for MA biosynthesis in graminaceous plants, suggesting its critical role in regulating iron uptake and homeostasis. There is a broad consensus that NAS is ubiquitously present in higher plants, though the number of encoding genes was limited in rice and *Arabidopsis*[31,34]. However, nine *NAS* genes were identified in barley by a combined screening strategy, indicating that NAS proteins may be encoded by a gene family and providing a possible link between the number of *NAS* genes and iron uptake strength [30]. In the previous study, due to unavailability of maize genomic sequence, only three *ZmNAS* were identified by screening a genomic library, though five ZmNAS proteins was predicted by western analysis [33]. Recently, many gene families were identified in maize by genomic mining, and it was also suggested that relatively more family members existed in maize than in another cereal crop, rice [38-41]. In our study, nine *ZmNAS* genes were systematically identified and characterized through genome wide analysis using the current version of maize inbred line B73 genome database. It is known that cereal genome undergoes two rounds of whole genome duplications associated with genome evolution. The fist occurred in all cereals before the specification of rice, sorghum and maize, whereas the second take place specifically in the lineage leading to maize [42]. Therefore, it is not surprising to identify more genes encoding NAS in maize than in rice. Besides, the increasing biomass and enhanced iron uptake and restoration features may be another driving force for the evolution and duplication of NAS in maize and barley. It was also interesting to find that NASs from graminaceous plants were divided into two classes by phylogenetic analysis, and relative more members were existed in class I in maize and barley than in rice. It was suggested that approximately one fourth of the genes in the maize genome possess closely related paralogs resulted from the genome duplication [43]. We found the class I *ZmNAS* genes duplicated as paralogs, and localized at duplicated region of maize genome, suggesting possible functional redundancy between them. Unlike class I, ZmNAS3, ZmNAS4 and ZmNAS5 share relatively lower identity, indicating a possibility of functional divergence between them. Interestingly, the paralogs, ZmNAS2;1 and ZmNAS2;2, are consisted of two full length NAS domain in tandem repeated. It was previously reported bacterium expressed ZmNAS2 (ZmNAS2;1) exhibited no NAS activity [33], though expression analysis revealed that *ZmNAS2;1/2;2* accumulated in roots and stems, and responded to fluctuated environmental iron status. Anyway, *in vivo* evidence are necessary to exclude (or confirm) the possibility they are not pseudogenes.

### Cytoplasm localization of ZmNAS

It can be assumed that the subcellular localization of NAS may affect NA compartmentalization in plant cell, and thus regulate the downstream utilizing of NA as an iron chelator or a precursor of MAs. It has been reported “particular vesicles” formed in the Fe-deficient barley root cells, which was suggested as the sites secreting MAs [44]. Pervious study showed ZmNAS1 (ZmNAS1;1) and ZmNAS2 (ZmNAS2;1) located to spot organelles in the cytoplasm, while ZmNAS3 distributed throughout the cytoplasm. The spot organelles were suggested as vesicles derived from the endoplasmic reticulum, which was thought to be the place for MAs synthesis [33]. In our study, the subcellular localization of each ZmNAS was determined by transient expressing the GFP fusion proteins in *Arabidopsis* mesophyll protoplasts (Figure 3) and onion epidermal cells (Additional file 3). Unexpected, all ZmNASs were localized at cytoplasm, suggesting that the N-terminal variable domain has little effect on subcellular localization. Since it is generally considered that over accumulation of the GFP fusion protein may lead to spot-like localization, the distinct results obtained between the present and pervious study may due to different transcription strength of the GFP fusion protein. Because the spot-like organelles in cytoplasm were not characterized in detail, further study concerning the subcellular localization of NAS family proteins should be applied by alternative methods, such as immunofluorescence.

### The complementary expression patterns of class I and class II *ZmNAS* genes links to their specific physiological functions

To date, the underlying mechanisms regulating iron uptake and translocation in plants are still not well understood, as well as the delicate transcriptional regulatory network involved in response to fluctuating environmental iron status. It has been reported the genes in strategy II Fe uptake system, such as *YS1/YSL*[21,45], *NAS*[6,33,34], *NAAT*[7], *DAMS*[8] and *TOM1* (a MAs efflux transporter) [46], were strongly induced under Fe deficiency, while those associated with metal detoxification were stimulated in response excessive environmental Fe [47]. Since the NA concentrations in tomato increase in response to Fe overload [48], arose the possibility that NA may play a critical role in regulating the balance between acquisition of environmental Fe and detoxification of excessive intracellular Fe. Therefore, it would be worthy to determine the response of *ZmNAS* genes to fluctuated environmental Fe status. It has been showed that the expression of *OsNAS1* and *OsNAS2* were increased in both roots and leaves under Fe deficiency, while that of *OsNAS3* was decreased in leaves and induced in roots in response to Fe deficiency [34]. Similar results were observed for *ZmNAS1* and *ZmNAS2*, though *ZmNAS3* was the first one reported to be repressed in roots under Fe deficiency [33].

**Figure 9:**
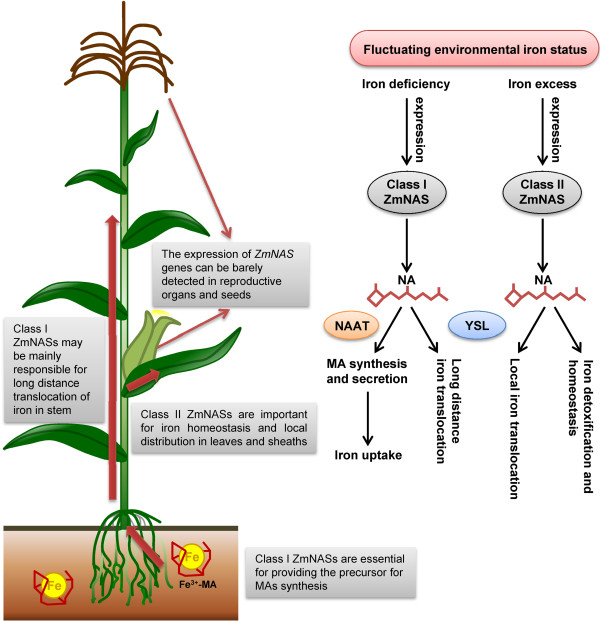
**Scheme showing the complementary expression patterns of class I and class II maize *NAS *genes link to their specific physiological functions.** The left panel shows the schematic diagram of putative physiological functions of ZmNASs during iron uptake and translocation of maize plant. The right panel shows the hypothetical model regulate the equilibrium between iron acquisition and homeostasis by establishing a complementary expression pattern of class I and class II maize *NAS* genes.

In our study, a comprehensive expression pattern of nine maize *NAS* genes were obtained based on the compilation of real-time RT-PCR and histological data. The class I *ZmNAS* genes accumulated significantly in roots and stems, while class II *ZmNAS* genes show divergence expression profiles: *ZmNAS3* and *ZmNAS4* expressed predominately in leaves and sheaths, while *ZmNAS5* accumulated mainly in stems and relatively lower in leaves and sheaths. Moreover, the class I *ZmNAS* genes were dramatically induced in both roots and shoots under Fe deficiency, but were repressed in response to Fe excess. In contrast, the expression of class II genes were down regulated under Fe deficient conditions, while that were retained during excessive Fe conditions. The complementary expression patterns of class I and class II *ZmNAS* genes suggested that maintaining high levels of NA in specific organs is essential for overcoming fluctuating iron status, and raises a model concerning their physiological roles in regulating Fe uptake and homeostasis. We hypothesize that the class I ZmNAS may mainly responsible for providing the precursor for MAs synthesis and long distance translocation of Fe in stem, while the class II ZmNAS produce NA for local distribution of Fe in leaf and sheath and detoxification of excess intracellular Fe (Figure 9). To verify this hypothesis, the histological localization of *ZmNAS1;1/1;2* and *ZmNAS3* were studied using *in situ* hybridization. The expression of *ZmNAS1;1/1;2*, two members in class I, was observed in cortex and stele of roots, while no signal was detected in shoots under Fe sufficient conditions. With Fe deficiency, *ZmNAS1;1/1;2* accumulation extended to epidermis associated with increasing demand for synthesizing and secreting MAs, indicating the class I ZmNASs are essential for providing precursor for phytosiderophore synthesis. *ZmNAS3* accumulated in leaf primordia, axillary meristems and mesophyll cells, suggesting a role for class II genes in local translocation of Fe, especially in developing organs. Since NA also chelate other metals, the expression of *ZmNASs* in response to Zn excess and Zn/Cu/Mn deficiency were examined. We found that the class I *ZmNAS* genes were induced under Zn deficiency, while they were repressed under Zn excess, and Cu/Mn deficiency. These results suggested that the increasing accumulation of class I *ZmNAS* genes in roots under changing environmental metal status may have selectivity for Fe and Zn. In addition, the class II genes were up-regulated under excess Zn, indicating that they may be essential for detoxification of excessive metal ions other than Fe.

### Biofortification of maize with high level of bioavailable Fe and Zn

Micronutrient deficiencies are mainly responsible for “hidden hunger”. In particular, the anemia caused by iron-deficiency is a prevalent nutrient problem in developing countries [49]. Maize is a major cereal crop for food supply and feed industry worldwide, though the lack of bioavailable Fe in corn can barely meet the demand. Therefore, addition essential metal elements were usually added in feeds to fulfil daily needs of animals. Alternatively, transgenic approaches can be applied to biofortificate the micronutrient content of crop plants. In the past, efforts were made in overexpressing *ferritin* from soybean and *Phaseolus vulgaris* in rice, and the Fe content was increased up to 3 and 2 fold [50,51]. Recently, NAS was chosen as a new candidate for improving micronutrient content. It was showed that activation of *OsNAS3* led to enhanced bioavailable Fe and Zn [27]. Similar result was obtained for *OsNAS1* and *OsNAS2*. Endosperm specific overexpression of *OsNAS1* enhance the Fe and Zn content up to 1.45 and 1.55 fold in unpolished grains, respectively [52]. Likewise, the Fe content in seeds of *OsNAS2*-activated rice was 3 fold higher than wild type [53]. Moreover, it was found that endosperm specific expression of *OsNAS1* could avoid negative effects on agronomic performance caused by constitutively overexpression [52], which suggested the original expression profile of *NAS* is essential for Fe homeostasis and thus affects plant growth. Therefore, the temporal and spatial RNA accumulation patterns of *ZmNAS* genes detected in this study may provide a delicate strategy to biofortificate maize with increased bioavailable iron.

## Conclusions

In this study, nine *NAS* genes in maize were identified by genomic mining. According to the evolutionary relationship of NAS from maize, barley, rice and *Arabidopsis*, ZmNAS and HvNAS can be subgrouped into two classes. Moreover, the temporal and spatial RNA accumulation patterns of *ZmNAS* genes were investigated in various organs including developing seeds, which further support the classification of *ZmNAS* gene family. Histochemical localizations of the *ZmNAS1;1/1;2* and *ZmNAS3*, which belongs to class I and class II, were determined by *in situ* hybridization. The complementary expression patterns of *ZmNAS* genes indicate maintaining sufficient NA is essential for overcoming fluctuating iron status. It was also hypothesized that the class I ZmNAS may be mainly responsible for supporting the precursor for MAs synthesis and long distance translocation of Fe, while the class II ZmNAS produce NA for local distribution of Fe and detoxification of excess intracellular Fe. These results provide significant insights into the molecular bases of ZmNAS in balancing iron uptake, translocation and homeostasis.

## Methods

### Plant materials

Maize inbred line B73 was surface-sterilized and germinated in vermiculite for 12 days in a greenhouse at 28°C. Then the seedlings were transferred into culture boxes and hydroponically grown to three-leaf stage in Hoagland nutrient solution. For metal-deficient treatment, the seedlings were transferred to Hoagland solution lacking indicated metals. For Fe and Zn excess treatment, 500 μM Fe^3+^-EDTA and 200 μM ZnSO_4_ were used. The shoots and roots from treated seedlings were sampled at indicated times and immediately frozen in liquid nitrogen and stored at −80°C until use. To detect the histochemical localization of *ZmNAS*, the samples were collected from 96 h treated seedlings and fixated in FAA.

### Identification of maize *NAS* genes

The sequences encoding putative NAS family members were identified using the TBLASTN program from the MaizeSequence database (http://www.maizesequence.org), using the protein sequence of previously identified ZmNAS1 as a query. The threshold of e-value and score for TBLASTN was set at 1e^-80^ and 600, respectively. In order to confirm the predicted genes encode ZmNASs, the protein sequences were searched in the Pfam database (http://pfam.sanger.ac.uk). In addition, full length coding cDNA sequences of all *ZmNAS* genes were further confirmed by cloning and sequencing. The primers used for cloning *ZmNAS* genes were listed in Additional file 4.

### Sequence alignment and phylogenetic tree construction

The deduced protein sequences of ZmNAS proteins were aligned with AtNAS1 and OsNAS1 using ClustalX 2.0.5 program. The phylogenetic tree was constructed with NAS proteins from Maize (Zm), Barley (Hv), Rice (Os), *Arabidopsis thaliana* (At) and *Solanum lycopersicum* (chlN) using the neighbor-joining method in MEGA 4.0 software. The proteins and their accession numbers used for alignment and phylogenetic tree construction are as follows: ZmNAS1;1 [MaizeSequence:GRMZM2G385200], ZmNAS1;2 [MaizeSequence:GRMZM2G312481], ZmNAS2;1 [MaizeSequence:GRMZM2G030036], ZmNAS2;2 [MaizeSequence:GRMZM2G124785], ZmNAS3 [MaizeSequence:GRMZM2G478568], ZmNAS4 [MaizeSequence:GRMZM2G439195], ZmNAS5 [MaizeSequence:GRMZM2G050108], ZmNAS6;1 [MaizeSequence:GRMZM2G704488], and ZmNAS6;2 [MaizeSequence:AC233955.1_FGT003] from Maize (*Zea mays*); NASHOR1 [GenBank:AF136941], NASHOR2 [GenBank:AF136942], HvNAS1 [GenBank:AB010086], HvNAS2 [GenBank:AB011265], HvNAS3 [GenBank:AB011264], HvNAS4 [GenBank:AB011266], HvNAS5 [GenBank:AB011268], HvNAS6 [GenBank:AB011269] and HvNAS7 [GenBank:AB019525] from barley (*Hordeum vulgare*), OsNAS1 [GenBank:AB021746], OsNAS2 [GenBank:AB023818] and OsNAS3 [GenBank:AB023819] from rice (*Oryza sativa*); AtNAS1 [GenBank:NM_120577], AtNAS2 [GenBank:NM_124990], AtNAS3 [GenBank:NM_100794] and AtNAS4 [GenBank:NM_104521] from *Arabidopsis thaliana*; chlN [GenBank:AJ242045] from *Lycopersicon esculen-tum*.

### RNA isolation and real-timeRT-PCR analysis

Total RNA was isolated using TRIzol reagent according to the manufacturer’s instructions (Invitrogen) Genomic DNA contaminants were removed from RNA samples using DNaseI (NEB). The amount and quality of the total RNA was confirmed by electrophoresis in 1% formamide agarose gel. Approximately 2 μg of total RNA was reverse transcribed to cDNA in 20 μL reaction using oligo-dT and M-MLV reverse transcriptase (Fermentas). Real-time PCR primers were designed to amplify a 100–200 bp fragments in untranslated regions. All primers were designed for 60°C annealing and their sequences are as follows: *ZmNAS1;1/1;2*, 5'-GAGGAGATGGCGACCACGACAGAGC-3^′^ and 5^′^-AGAAGTGCATGAGAAATTCAGAAGC-3^′^; *ZmNAS2;1/2;2*, 5^′^-AGTGCTGCAAGATGGAGGCGAAC-3^′^ and 5^′^-AGTTACACGAGAGATTGAAACAG-3^′^; *ZmNAS3*, 5^′^-GGCTCACCAGAAGATGGAGGAG-3^′^ and 5^′^-TCACGCATGTGGTGTAGACACG-3^′^; *ZmNAS4*, 5^′^-CACGGCACACACCACAAGCAACAAG-3^′^ and 5^′^-ATCCATGCGGTGTGGGCACATAGAC-3^′^; *ZmNAS5*, 5^′^-ACCGGCGTCCTCGCTTTCTTGTC-3^′^ and 5^′^-ACGATATGCGGATGCGGTCAGCCAG-3^′^; *ZmNAS6;1/6;2* 5^′^-CTTGCAGCACCAAGTTGTCGAAC-3^′^ and 5^′^-CATGGAAGTTGTGGTTGCTACGG-3^′^; *ZmActin1*, 5^′^-ATGTTTCCTGGGATTGCCGAT-3^′^ and 5^′^-CCAGTTTCGTCATACTCTCCCTTG-3^′^. Real-time RT-PCR was performed with an ABI7500 cycler (Applied Biosystems) using the SYBR Premix Ex-Taq master Mix (TakaRa). Reactions were performed in a total volume of 20 μL with 2 μL of 20×diluted cDNA, 0.2 mM gene-specific primers and 10 μL of 2×SYBR premix. The PCR conditions were initial denaturation at 95°C for 30 s, followed by 40 cycles composed of 5 s denaturation at 95°C and 34 s of annealing/extension at 60°C. To verify specific amplification, melting-curve analysis was performed and the PCR products were separated by electrophoresis and sequenced. Data were analyzed with the ABI7500 software (version 2.0.5) via the ΔΔC_T_ method, and the expression levels of *ZmActin1* were used as an internal control. For all real-time PCR analysis, two biological replicates were used and three technical replicates were performed for each biological replicate.

### Subcellular localization of the ZmNAS-GFP fusion protein

The coding region of GFP was amplified with the following primers, 5^′^- CTCGAGGGATCCCCGGGAATTCCATGGAGCTCGGTACCTCTAGAATGGTGAGCAAGGGCGAG 3^′^ and 5^′^- TACTAGTTTACTTGTACAGCTCGTCCATGC -3^′^, and the resulting fragment was cloned into the *Xho*I-*Xba*I sites of plant expression vector pRTL2 to generate the plasmid pRTL2GFP. To examine the subcellular localization of ZmNAS proteins, the entire coding region of each gene were cloned in between the cauliflower mosaic virus 35S promoter and GFP of pRTL2GFP vector. The primers used for cloning coding regions of *ZmNAS* genes are listed in Additional file 5. The *ZmNAS*-*GFP* fusion constructs were transformed into *Arabidopsis* mesophyll protoplasts as described previously [54]. After incubation in the dark at 26°C for 14 h, the fluorescence was examined using a confocal microscope (LSM700; Carl Zeiss).

### mRNA *in situ* hybridization

*In situ* hybridization was performed as described previously [55] with slight modifications. The shoots and roots were collected from Fe-deficient and excessive treated seedlings and fixed in FAA solution containing 50% ethanol, 5% acetic acid, and 3.7% formaldehyde. To examine the mRNA localization of *ZmNAS1;1/1;2* and *ZmNAS3*, the specific sequences corresponding to the 3^′^-region of mRNA were amplified with the following primers, *ZmNAS1;1/1;2*, 5^′^- TTCCATGGATCGTCGATCCTGAGGACATTCGTC -3^′^ and 5^′^- TTACTAGTAGAAGTGCATGAGAAATTCAGAAGC -3^′^; *ZmNAS3*, 5^′^- TTAAGCTTACTCCGTCATCATCGCCCGCAAGC -3^′^ and 5^′^- TTACTAGTAAATTAGGCCAGCCTGTTCGCTC -3^′^; The PCR products were cloned into the vector pEasy-T3 to generate pEasy-NAS1ISH and pEasy-NAS3ISH, then the resulting plasmids were sequenced and linerized. The Digoxigenin-labeled sense and antisense RNA probes were *in vitro* transcripted by T7 and SP6 RNA polymerase (Roche) using *Spe*I and *Nco*I digested pEasy-NAS1ISH, and *Spe*I and *Hind*III digested pEasy-NAS3ISH, respectively. The hybridization was performed with a probe concentration of 0.4 ng μL^-1^ at 55°C in a wet chamber. The enzyme-catalyzed insoluble purple signal was visualized with a Zeiss Axioscop 4.0 microscope and photographed (Zeiss Mrc5, Germany).

## Abbreviations

NAS: Nicotianamine synthase; MAs: Mugineic acid; PS: Phytosiderophores; DMA: 2^′^-Deoxymugineic acid; NAAT: Nicotianamine aminotransferase; DMAS: Deoxymugineic acid synthase; YSL: Yellow strip like transporter; GFP: Green fluorescent protein.

## Competing interests

The authors declare that they have no competing interests.

## Authors’ contributions

XJZ and RMC conceived and designed the research. XJZ performed the bioinformatics analysis, gene cloning, real-time RT-PCR and *in situ* hybridization. SZL prepare the plant materials and carried out subcellular localization experiments. QQZ assisted in gene cloning and plasmid construction. XQL and SJZ collected the tissues for temporal and spatial expression analysis. CS helped in bioinformatics analysis. XJZ analysed the data and drafted the manuscript. RMC,CYZ and YLF contributed to revisions of the manuscript. All authors read and approved the final manuscript.

## Supplementary Material

Additional file 1 **The amino acid sequence alignment of class I maize NAS.** A pdf file shows the amino acid sequence alignment of maize NAS1;1/1;2/6;1/6;2 (**A**) and NAS2;1/2;2 (**B**). Click here for file

Additional file 2 **The cDNA sequence alignment of class I maize *NAS.*** A pdf file shows the cDNA sequence alignment of maize *NAS1;1*/*1;2*/*6;1*/*6;2* (**A**) and *NAS2;1*/*2;2* (**B**). The blue and red arrow indicates the translation start site and stop codon, respectively. Click here for file

Additional file 3 **Subcellular localization of ZmNAS-GFP fusion proteins in onion epidermal cells.** A pdf file shows the Subcellular localization of ZmNAS-GFP fusion proteins in onion epidermal cells. The coding regions of ZmNAS genes were C-terminal fused with GFP and were transiently expressed in onion epidermal cells driven by micro-particle bombardment. The images were obtained by a confocal microscope, and the cytoplasm localization of GFP is used as a control. The scale bar represents 50 μm. Click here for file

Additional file 4 **Primers used for cloning *ZmNAS *genes.** Excel document contains primer sequences used for cloning *ZmNAS* genes. Click here for file

Additional file 5 **Primers used for cloning the coding region of *ZmNAS *in subcellular localization assay.** Excel document contains primer sequences used for cloning the coding region of *ZmNAS* in subcellular localization assay. Click here for file
