# Supplementary material for: Genome-wide identification, classification and expression profiling of nicotianamine synthase (NAS) gene family in maize
Source: BMC Genomics. 2013 Apr 10;14:238. doi: 10.1186/1471-2164-14-238 (PMC3637603; doi:10.1186/1471-2164-14-238)
Supplement: Additional file 2 — The cDNA sequence alignment of class I maize NAS. A pdf file shows the cDNA sequence alignment of maize NAS1;1/1;2/6;1/6;2 (A) and NAS2;1/2;2 (B). The blue and red arrow indicates the translation start site and stop codon, respectively. [file 1471-2164-14-238-S2.pdf]

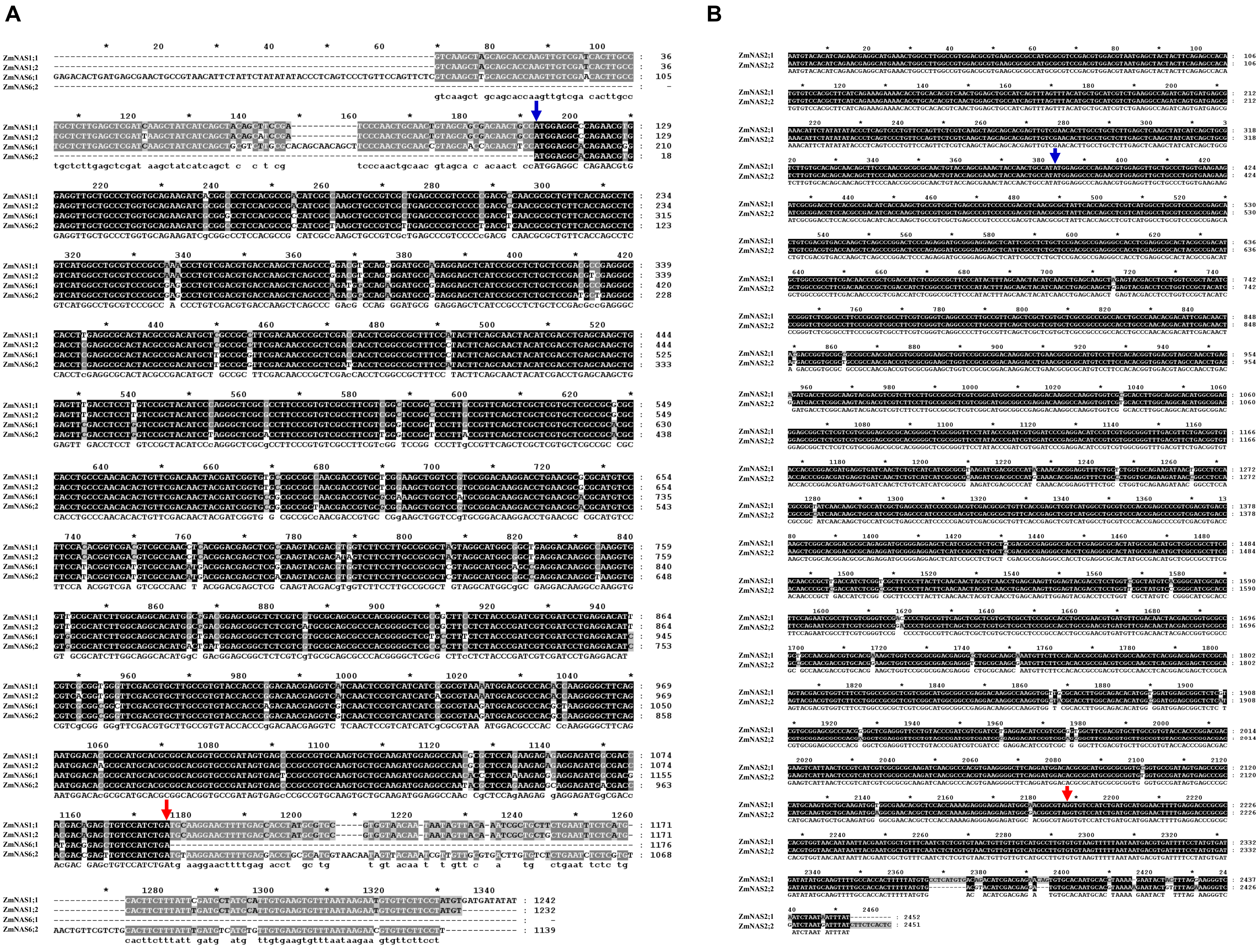
A
ZmNAS1;1
ZmNAS1;2
ZmNAS6;1
ZmNAS6;2
B
ZmNAS2;1
ZmNAS2;2
