# Supplementary figures and images for: Genome-wide identification, classification and expression profiling of nicotianamine synthase (NAS) gene family in maize
Source: BMC Genomics. 2013 Apr 10;14:238. doi: 10.1186/1471-2164-14-238 (PMC3637603; doi:10.1186/1471-2164-14-238)

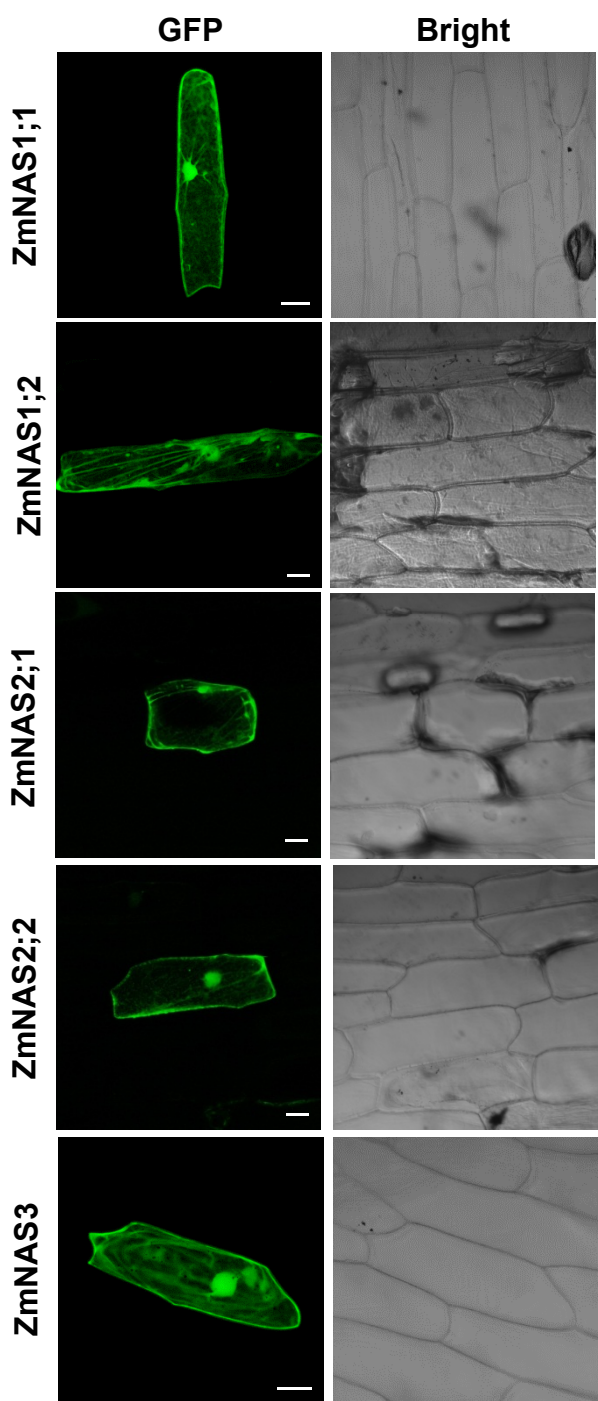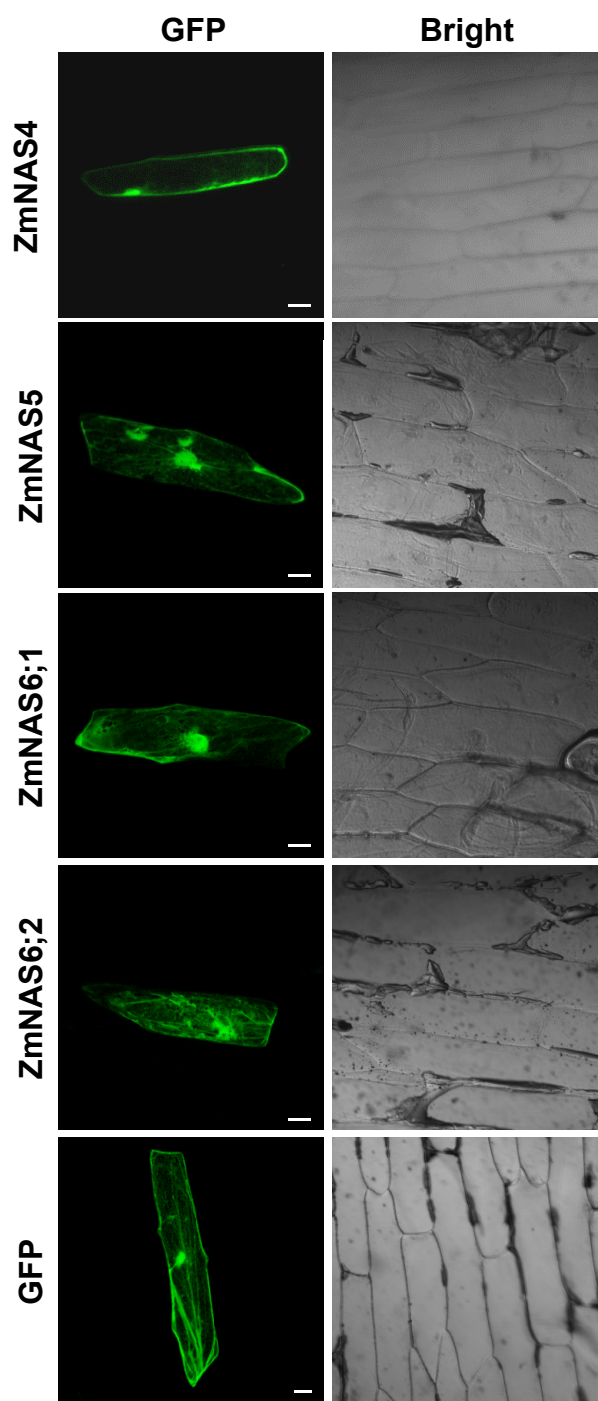

Supplement: Additional file 3 — Subcellular localization of ZmNAS-GFP fusion proteins in onion epidermal cells. A pdf file shows the Subcellular localization of ZmNAS-GFP fusion proteins in onion epidermal cells. The coding regions of ZmNAS genes were C-terminal fused with GFP and were transiently expressed in onion epidermal cells driven by micro-particle bombardment. The images were obtained by a confocal microscope, and the cytoplasm localization of GFP is used as a control. The scale bar represents 50 μm. [file 1471-2164-14-238-S3.pdf]
